# Supplementary material for: Discovery of New Markers for Haemogenic Endothelium and Haematopoietic Progenitors in the Mouse Yolk Sac
Source: J Dev Biol. 2026 Jan 6;14(1):4. doi: 10.3390/jdb14010004 (PMC12821522; doi:10.3390/jdb14010004)
Supplement: Supplementary file 1 [file jdb-14-00004-s001.zip › Supplementary Figures.pdf]

Supplementary Figure S1

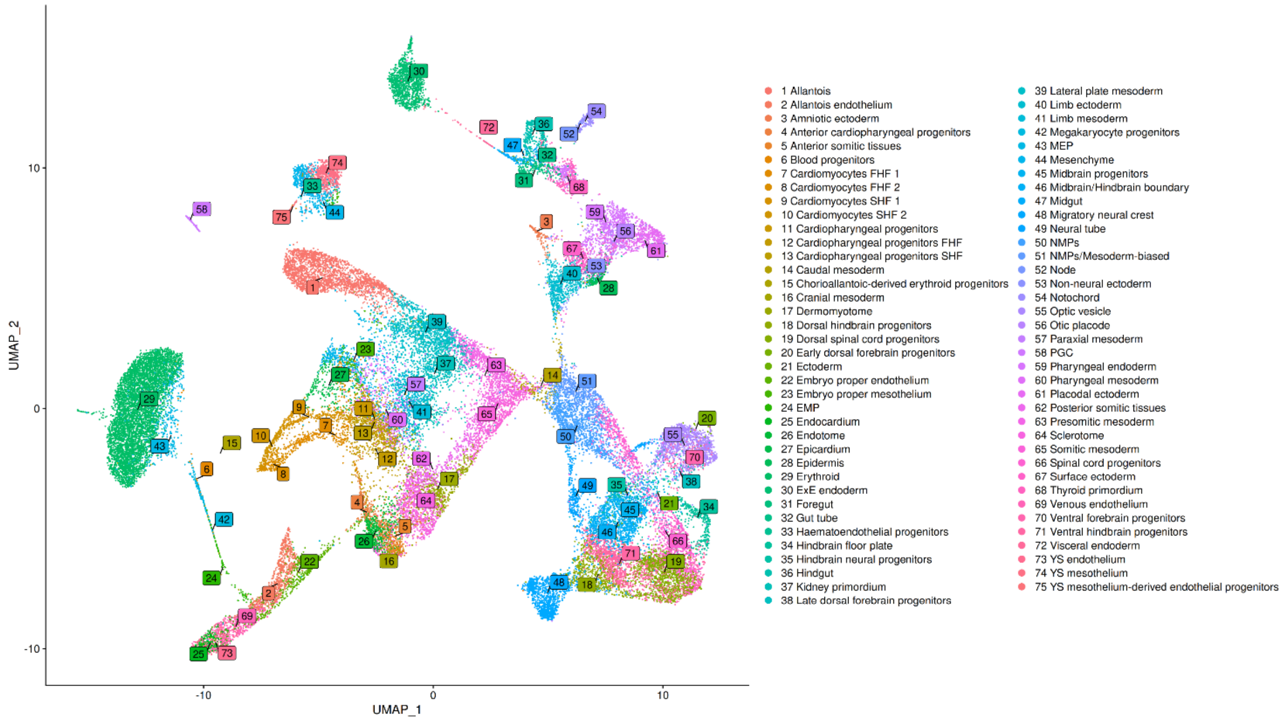

Fig S1. Cluster annotation of E8.5 mouse scRNAseq data in E-MTAB-11763.

UMAP of the E8.5 cells in E-MTAB-11763 dataset, labelled with the cluster annotations provided by the authors of the original publication (Goh et al., 2023).

Supplementary Figure S2

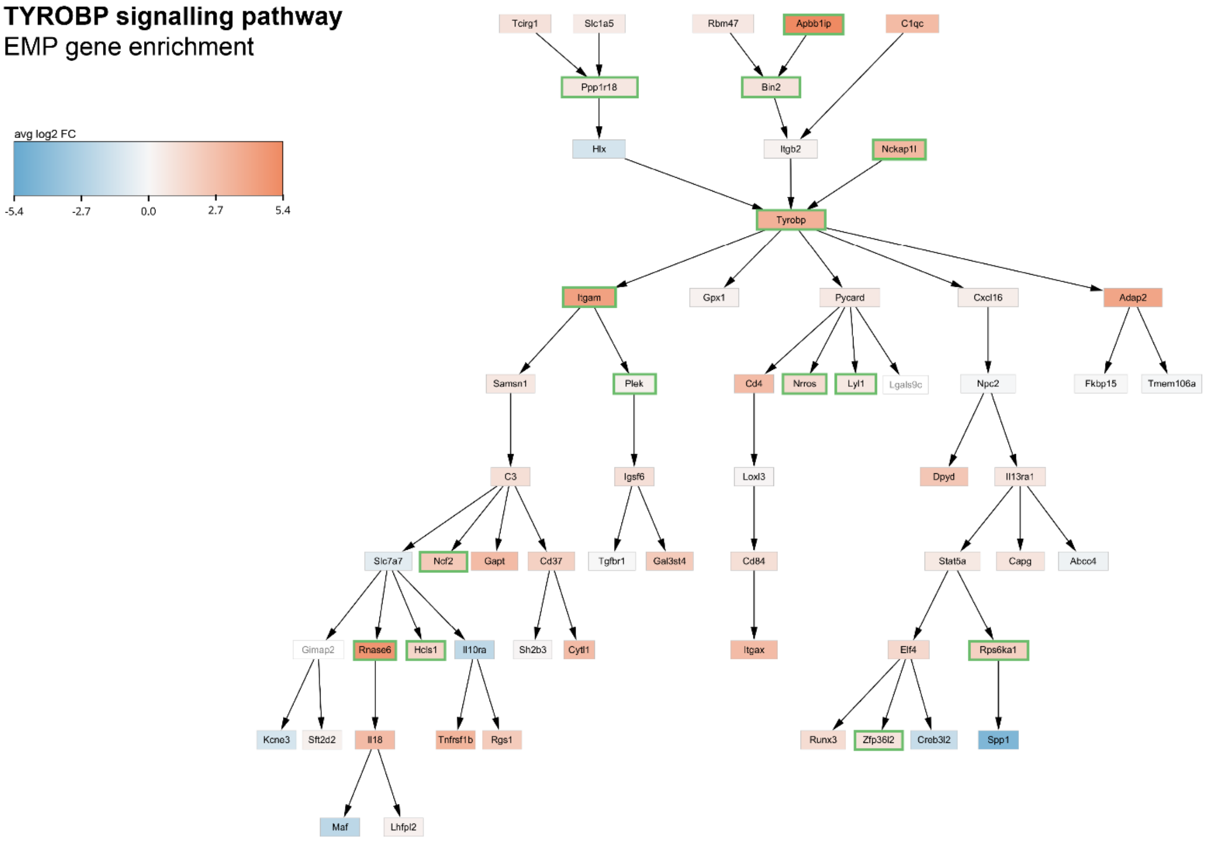

Fig S2. The TYROBP signalling pathway is enriched in emerging EMPs.

TYROBP pathway genes enriched in EMPs compared to haemato-vascular clusters from both yolk sac and embryo. The box colour indicates the average log2 fold change. A green border indicates significant enrichment in EMPs.

Supplementary Figure S3

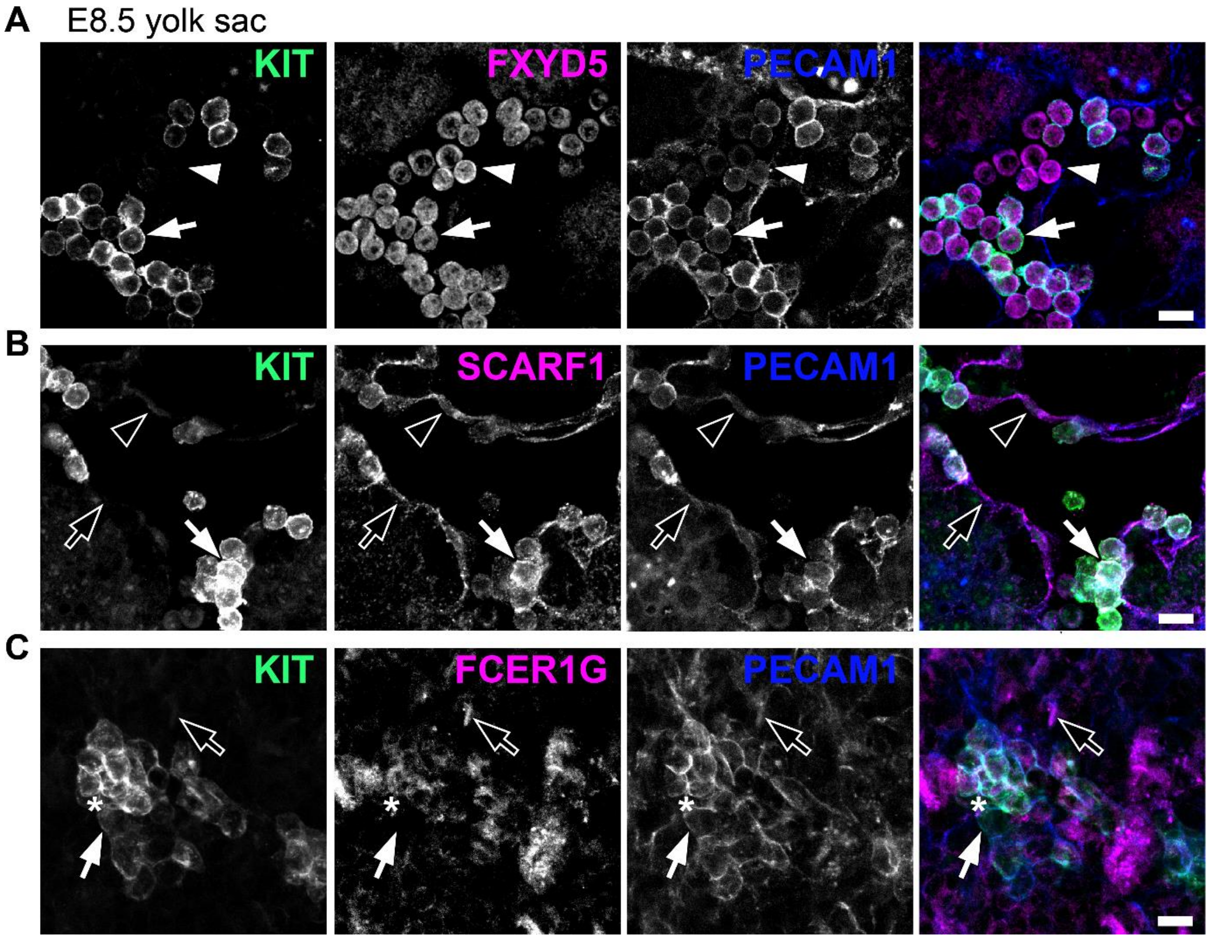

Fig S3. Immunostaining validation of new markers for haemogenic endothelial cells and EMPs.

A-C Immunofluorescence staining of E8.5 yolk sac for the EMP marker KIT together with PECAM1 and FXYD5, SCARF1 or FCER1G. Emerging EMPs are indicated with arrows (round KIT+ PECAM1+ cells), presumed blood (myeloid) progenitors with arrowheads (round KIT- PECAM1low cells), endothelial cells with empty arrows (flat KIT- PECAM1+ cells), haemogenic endothelial cells with empty arrowheads (flat KIT+ PECAM1+ cells) and mature EMPs with asterisk (round KIT+ PECAM1+ FCER1G+ cells). Scale bars: 50 µm; n = 3 embryos.
